# The Poplar Hospital

**Published:** 1889-12-07

**Authors:** 


### THE HOSPITAL WORKSHOP.

### No. XVII.—THE POPLAR HOSPITAL FOR ACCIDENTS.

(BY OUR SPECIAL COMMISSIONER.)

This hospital lies in the heart of a crowded and busy district. The building, originally a custom-house, is of red brick, and still wears a decidedly official look. It stands close to the main entrance of the East India Docks, a few minutes' walk from Blackwall railway station. This is the first hospital met with in sailing up the Thames, and accidents are brought hither from the East and West India, the Royal Albert, the Tilbury, and other lesser docks. Many trades also flourish in the district, such as the shipbuilding yards of Green's and of the Yarrow; numerous factories, of which the Thames Iron Works may be taken as a type; besides engineers' workshops, chemical works, and warehouses galore. On the back of its report the committee print a few lines which presents the scope of its operations in a nutshell: "The hospital is situated close to the dock gates and great engineering works, in the very spot where appalling accidents are most rife. It is more than two miles distant from the nearest general hospital. Time and transport, always painful, are often vital considerations." The hospital alluded to is the "London," and many cases have to be sent on thither, a journey which must of necessity add to the risks of sufferers.* The daily average of occupied beds at Poplar is thirty-five. After what has been said about the nature of the district, the reader will not be surprised to hear that such a provision entirely fails to meet the requirements of the case. The wards are always crowded. Many serious cases have to be turned away from the doors, and patients are being constantly hurried off to make room for fresh comers.

A large proportion of applicants come from the riverside. One of the commonest accidents is for the "docker" to tumble down the hold of the vessel on which he is at work. In the report for the year 1888 short notes are given of two out of many such cases. A kind of dramatic interest is raised by the fact that both occurred together, and one was fatal.

"November 13.—A. O'K.—Fell 25ft. into ship's hold; was insensible on admission, suffering from concussion of the brain; fractured collar bone, and compound fracture of bones of leg. Made an uninterruptedly good recovery, leaving hospital on January 18, 1889."

"November 13.—W.C.—Fell down the same hold, and at the same time as previous case, but unfortunately one of the hatches fell across his abdomen, causing rupture of the liver and other damage. He died within twelve hours after admission."

Another fruitful source of accident is the falling of "ballast," which crushes the men against the side of the ship. A good many cases are sent in from the dry docks, especially in foggy weather. During the late strikes, a number of the casual men—"blacklegs," as they are called—were brought to the hospital suffering from various mishaps met with through not having been used to the work.

As may be imagined, the injuries are often very severe. That fact may be inferred from a statement in the report to the effect that "eleven persons succumbed to their injuries while being conveyed to the hospital." Of the cases that terminate fatally, many die within twenty-four hours of the time of admission. Last year there were forty-seven deaths, of which fourteen were due to fractures of the skull. Speaking broadly, results of treatment are extremely good, due in great measure to the generally robust condition of patients. Indeed, some of the cures are marvellous considering the exposure and rough travelling which sailors often have to undergo before they can be placed under skilled treatment. One man, for instance, fell on deck from the yard arm, and sustained a compound fracture of the thigh besides severe head injuries. It was four days before the ship reached port, and he was carried to the Poplar Hospital, well nigh dead, yet he made a rapid and perfect recovery.

An analysis of cases for last year offers some interesting

*The new branch hospital of the Dreadnought Seamen's Hospital, Greenwich, now being erected on a site in the docks, will remove this risk very shortly.—Ed.

and suggestive facts. There were no less than 990 breaks of various bones; 78 of them compound; and 258 treated in the wards; 231 were fractures of bones of the arm; 158 of ribs; 124 of collar bones; and 33 of the skull. Inflammation after injury accounts for 620 cases, and 6,531 are credited to "wounds, contusions, lacerations, and sprains."

By far the greater number of these cases are treated as out-patients. That department has lately increased by leaps and bounds, and is conducted in the basement, below the level of the street. Altogether your commissioner can safely say it is about the worst specimen of out-patient accommodation it has ever been his lot to inspect. Dark, badly ventilated, apparently damp, dismal, cramped, the place must be unhealthy and depressing for all concerned. Yet there is an immense amount of good work done there by the two resident officers, who practically attend to the whole department, in addition to the care of in-patients. When it is remembered that cases of more or less severity are dropping in all day long and often throughout the night as well, it will be tolerably clear that the two house surgeons must be taxed to the utmost of their energies. It is too much the fashion of committees to overwork and underpay their medical officers. No other profession offers the spectacle of highly-trained men after years of expensive special education doing laborious work at the wages of unskilled labour. Hospitals, also, let it be recalled to mind, get the services of their honorary staff free, gratis, and for nothing.

After these few remarks, addressed to charitable committees generally, and not in the least particularly to the Poplar board, which includes the names of several distinguished philanthropists, let us turn again to the report. Burns and scalds mount up to 400, the greater part of the patients being children who have been left at home by their parents. A great many children are also brought here who have been run over in the streets of the crowded districts that have sprung up round the docks. There are 236 cases of bites of various animals, machinery accidents are common, and very many are sent in from the railways. The proportion of medical cases is small, 410 to 10,320 surgical. They include alcoholism, epilepsy, diarrhœa, rheumatism, and skin diseases, but, curiously enough, no special mention is made of tropical affections, which one would expect to figure under this heading.

The first ward visited was "The Fowler." It is a homely room, with polished floor, running from front to back of the building, and lighted by windows at each end. The side opposite the entrance opens into an added ward. One bed is occupied by a boy of sixteen, who was caught between the buffer and chains of some trucks while at work on a railway. He has sustained severe internal injuries, but, although the accident happened only a few days' since, he is able to muster up a smile when spoken to by the house surgeon. Near this patient is a man recovering from fracture of the base of the skull and arm broken just above the elbow. He is a dock labourer, and improved methods of treatment have probably saved his life, yet the injury to his head has left mischief behind—such as deafness and confusion of mind—that will most likely be permanent. While on this subject, another serious defect in the hospital accommodation may be pointed out. The proper treatment of this class of cases demands absolute quiet and isolation, but there is no ward available for such a purpose, so that a person suffering from fracture of the base of the skull may be lying between a couple of delirious and noisy patients. A small room at the top of the house is sometimes pressed into the service.

In this ward a swarthy-faced young Lascar from Bombay tells us he is in the P. and O. Company's service, and is now nearly well. In the next ward was pointed out an empty bed, lately occupied by another Lascar, Sala Cuteen, who had happily recovered from an irritable concussion of the brain. Foreigners of every kind find admission—for instance, French, Germans, Italians, Greeks, and other Europeans, with stray visitors from almost every known race in the world. Late visitors include a Madagascar chief, but, unfortunately, his name could not be ascertained. Another inmate, a Japanese, displayed an extraordinary quickness of apprehension, although he could not speak a single word of English. From simply watching the other patients play at draughts, he picked up in one evening sufficient knowledge of the game to beat everyone else in the ward next day. As to the surgical relationships of these foreigners, there is a kind of international language, independent of words, which makes itself understood upon such occasions. Suppose some "Sheik Mahomet" to come shivering to the hospital, clad in a thin and fantastic garb, and displaying a broken radius. It needs no interchange of Volapük, nor, for that matter, of Hindustanee and English, to enable the injured man to point to his helpless arm, and the house surgeon to feel the crepitus and put on the necessary splints and bandages.

Enough has been said to give readers an idea as to the class of accidents and the special value of the hospital. A word should be added as to the nurses' quarters at the top of the house. Some few of them, as we were told, have to sleep in a branch of the main dormitory running between the small room on the one side, where occasional noisy cases are lodged, and the children's ward on the other. Nothing beyond a simple statement of this fact is required. The state of the medical officers' rooms also leave much to be desired; they are deficient in comfort, and placed in a noisy part of the building.

The children's ward is on the top floor, and contains eight cots, besides three beds for adults. Ventilation appears to depend on the windows, running down the front of the ward, and on a fireplace at each end. At the time of our visit the atmosphere was unmistakably "stuffy." In short, the building was never meant for a hospital, and should be replaced by an enlarged and entirely new modern building. The institution is doing a very great deal of good work, but, mainly owing to its position in what is to greater London an unknown wilderness, has hitherto been allowed to struggle on in undeserved neglect. All treatment is free, and, since it is practically restricted to accidents, cannot be charged with those abuses with which many of the charities are now confronted. Lastly, the important fact should be mentioned that the hospital is to a large extent supported by the subscriptions and efforts of the working-men themselves. A special list of their contributions is appended to the report, but such a division is apt to be misleading, for many of the sums appearing in the general list after individual names are in reality made up jointly by employers and employed. The Poplar Hospital for Accidents is worthy of public confidence and increased support.

